# Development and Application of a Rapid Detection Technique for 2-Amino-1-methyl-6-phenyl-imidazo [4,5-b] Pyridine in Meat Products Based on Hydrogel-Molecular-Imprinting Electrochemical Sensing Technology

**DOI:** 10.3390/foods14081292

**Published:** 2025-04-08

**Authors:** Chunxiao Li, Xiaolei Zhao, Xiaofei Yin, Shuting Zhang, Jinxing He

**Affiliations:** Shandong Key Laboratory of Healthy Food Resources Exploration and Creation, School of Food Science and Engineering, Qilu University of Technology, Jinan 250353, China; 10431221143@stu.qlu.edu.cn (C.L.); zxl1989330@163.com (X.Z.); 202193070034@stu.qlu.edu.cn (X.Y.); 202195090011@stu.qlu.edu.cn (S.Z.)

**Keywords:** molecularly imprinted hydrogel, carcinogenic compounds, electrochemical analysis, heterocyclic amines

## Abstract

2-amino-1-methyl-6-phenyl-imidazo [4,5-b] pyridine (PhIP) is a harmful compound that is formed during the high-temperature processing of meat products, and the risk of cancer may be increased with the prolonged intake of foods containing high levels of PhIP. This study aimed to develop an innovative detection method specifically for PhIP in meat products. Utilizing hydrogel-molecular-imprinting electrochemical sensing technology, the preparation conditions of molecularly imprinted hydrogels (MIHs) were optimized. Consequently, a highly selective and rapid detection method for PhIP was successfully established, integrated with an electrochemical workstation. The results indicate that the prepared MIHs exhibit an excellent specific recognition performance for PhIP. The sensor exhibited a linear response within the concentration range of 1.0–200.0 ng/mL, with a detection limit of 0.07 ng/mL (S/N = 3) under optimized conditions. In addition, the accuracy and reliability of the method were verified by spiked recovery experiments, and the recoveries ranged from 75.9% to 108.8%, which demonstrated its high accuracy and potential for practical application.

## 1. Introduction

With the thriving development of the modern food industry, food safety issues have increasingly become the focus of public attention. They not only concern the health and wellbeing of everyone but also constitute an important topic in current international exchanges [1]. In daily life, meat and its products play a pivotal role, yet they also come with a series of potential health risks that cannot be ignored [2]. For instance, during the high-temperature processing of meat products, various harmful substances are prone to being generated, among which HCAs (heterocyclic amines) are the most representative class of compounds [3]. Notably, 2-amino-1-methyl-6-phenyl-imidazo [4,5-b] pyridine (PhIP), a type of HCA, is particularly common [4]. It typically forms in meat products rich in protein, existing at concentrations in the order of ng/g, and is most prevalent during chicken processing [5]. Studies have shown that PhIP can be converted into active metabolites in the human body, and the long-term consumption of food with high PhIP levels may increase the risk of cancer. The formation mechanism of PhIP is quite complex [6], being influenced by numerous factors, including the type and quality of raw meat, the method and duration of heat treatment, as well as temperature [7]. In light of this, the development of an efficient and sensitive detection method for PhIP is of crucial importance for ensuring food safety.

Currently, the commonly used detection methods for PhIP in meat products mainly include High-Performance Liquid Chromatography (HPLC) [8], Liquid Chromatography–Mass Spectrometry (LC–MS) [9], Gas Chromatography–Mass Spectrometry (GC–MS) [10], and Enzyme-Linked Immunosorbent Assay (ELISA) [11]. Although these methods can detect PhIP, they also have certain limitations in some aspects. For instance, methods such as HPLC and LC–MS, despite their high accuracy, involve complex sample pretreatment and analysis processes that require professional technicians to operate, and the entire analysis workflow is time consuming [12]. Additionally, the equipment required for these techniques is expensive, with high maintenance costs and stringent laboratory conditions. In contrast, while ELISA is relatively simple to operate, it has lower sensitivity and specificity, and is susceptible to interference from other sample matrices, which may affect the detection results. Therefore, there is an urgent need to develop a novel, efficient, and sensitive detection method for PhIP to overcome the shortcomings of existing technologies.

Since the 1990s, molecular imprinting technology (MIT) has undergone rapid development, particularly in the preparation of highly selective biomimetic recognition materials, achieving remarkable accomplishments [13]. This technology simulates the interactions between antigens and antibodies, as well as enzymes and substrates in nature, to produce polymer materials with recognition capabilities, known as molecular recognition technology. This is a specific molecular recognition method discovered by MIT. Among them, molecularly imprinted polymers (MIPs) are formed through the interaction between template molecules and functional monomers to create a three-dimensional network structure [14]. When the template molecules are removed, the resulting imprinted cavities can specifically recognize target molecules, enabling selective molecular recognition. This gives MIPs high selectivity, stability, and reusability [15]. However, traditional MIPs have limitations in terms of their preparation and application, such as an uneven distribution of recognition sites, harsh synthesis conditions, and low mass transfer efficiency, which restrict their development in practical applications [16]. To overcome these limitations, researchers have continuously explored new materials, among which hydrogels as carrier materials for molecular imprinting exhibit great potential. Hydrogels are polymeric materials with a three-dimensional network structure that can absorb and retain large amounts of water, exhibiting excellent biocompatibility, mechanical strength, flexibility, and a porous structure [17]. The application of hydrogels in molecular imprinting technology allows for the preparation of MIHs, which not only possess the high selectivity and stability of traditional MIPs but also optimize the distribution of recognition sites and enhance mass transfer efficiency due to the incorporation of hydrogels [18]. Additionally, the flexibility of hydrogels enables MIHs to adapt to complex reaction environments, thereby improving the sensitivity and accuracy of detection. Therefore, detection methods based on molecularly imprinted-hydrogel technology have broad application prospects in food safety testing.

Electrochemical detection is a technique that relies on changes in current or voltage during electrochemical reactions to detect target analytes [19]. Compared to other detection methods, electrochemical sensor technology can utilize various technical means, such as Cyclic Voltammetry (CV) and Differential Pulse Voltammetry (DPV) [20], to monitor subtle changes in current and voltage in real time. In particular, DPV technology enables the precise detection of the presence and concentration of target analytes, significantly enhancing detection sensitivity and making it particularly suitable for trace analyte detection [21]. Furthermore, electrochemical sensor technology offer advantages over other detection equipment (like LC–MS and GC–MS) in terms of its lower cost, ease of maintenance, suitability for rapid on-site detection, and operation by non-professionals [22]. Consequently, this have been widely applied in the field of food safety testing. In recent years, research on molecularly imprinted hydrogels has been conducted. For instance, studies have successfully prepared molecularly imprinted hydrogels for the selective extraction of tetracycline antibiotics in milk by optimizing parameters such as the ratio of template molecules to functional monomers, and utilized electrochemical sensor technology for detection [23]. This combination of molecularly imprinted hydrogels and electrochemical sensor technology not only verifies the high selectivity and stability of the hydrogels but also significantly boosts detection sensitivity. However, molecularly imprinted-hydrogel electrochemical sensors still face a number of challenges in practical applications. In order to obtain highly selective and stable molecularly imprinted hydrogels, the ratio of template molecules to functional monomers as well as other synthetic parameters need to be precisely controlled, which increases the complexity and cost of the preparation process. Although molecularly imprinted hydrogels are somewhat reusable, their properties may deteriorate after multiple uses.

We aimed to develop an electrochemical detection method utilizing molecularly imprinted-hydrogel technology for the rapid and precise identification of PhIP in meat products. This method primarily leverages the combination of the advantages of molecularly imprinted hydrogels and electrochemical sensor technology to achieve simple, rapid, and highly sensitive detection. Firstly, a polydopamine layer is formed on the surface of a glassy carbon electrode through electrodeposition. This step aims to enhance the adhesion of subsequent conductive materials and molecularly imprinted hydrogels on the electrode surface, while simultaneously improving the detection sensitivity. Next, the conductive material, comprising multi-walled carbon nanotubes, is modified onto the electrode surface to further enhance its conductivity, thereby further improving the detection sensitivity. Finally, the prepared MIHs are modified onto the electrode, and the target compound PhIP is detected through DPV.

This study presents an innovative integration of hydrogel molecular blotting and electrochemical sensing technologies. Our approach uniquely targets PhIP, a structurally intricate carcinogen, by optimizing hydrogel synthesis and modifying the polydopamine–carbon nanotube electrode. Moreover, our method necessitates fewer pretreatment steps compared to traditional techniques like HPLC, enhancing its efficiency. The materials employed in this sensor are not only more cost-effective but also readily accessible, thereby promoting its extensive application in research and other fields. Additionally, regarding practical applications, we have validated the practical utility of our sensor through testing on actual meat samples.

## 2. Materials and Methods

### 2.1. Materials and Equipment

The primary materials used in this experiment were: acrylamide, azobisisobutyronitrile, ethylene glycol dimethacrylate, potassium ferricyanide, and potassium ferrocyanide, all sourced from Aladdin Chemical Reagent Co., Ltd. (Shanghai, China); 2-amino-3-methyl-3H-imidazo[4,5-f]quinoline, α-methacrylic acid, and dopamine hydrochloride, obtained from Shanghai Macklin Biochemical Co., Ltd., Shanghai, China; 2-amino-1-methyl-6-phenylimidazo[4,5-b]pyridine, acquired from Shanghai Anpu Cuishi Standard Technical Service Co., Ltd., Shanghai, China; as well as 2-amino-3,8-dimethylimidazoquinoxaline and 3-amino-9-ethylcarbazole, purchased from Shanghai Yuanye Bio-Technology Co., Ltd., Shanghai, China. Additionally, the carboxylated multi-walled carbon nanotubes were sourced from Qingdao Henglide Graphite Co., Ltd., Qingdao, China. Concurrently, reagents such as potassium chloride, acetic acid, and sulfuric acid were also obtained from Sinopharm Chemical Reagent Co., Ltd. (Shanghai, China).

In terms of instrumentation, this experiment employed a UV–Vis spectrophotometer (model: YU-1901, manufactured by Beijing Puxi General Instrument Co., Ltd., Beijing, China) for measuring UV–Vis absorption spectra. All electrochemical measurements were conducted using the CHI760E electrochemical workstation (manufactured by Shanghai Chenhua Instrument Co., Ltd., Shanghai, China). During the measurements of CV and DPV, a traditional three-electrode system was adopted, with a glassy carbon electrode serving as the working electrode, an Ag/AgCl electrode as the reference electrode, and a Pt wire as the counter electrode.

### 2.2. Method

#### 2.2.1. Preparation of Molecularly Imprinted Hydrogels

**Figure 1:**
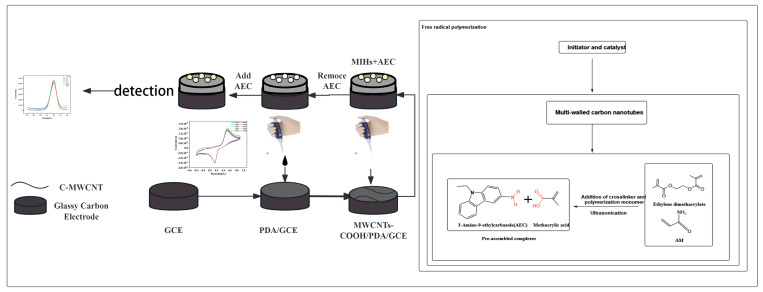
Flowchart of the preparation of molecularly imprinted-hydrogel electrochemical sensor.

Figure 1 shows the preparation process of the molecularly imprinted-hydrogel electrochemical sensor. Firstly, 0.5 mmol of the template molecule of 3-amino-9-ethylcarbazole (AEC) was accurately weighed and dissolved in a pre-configured solvent mixture of chloroform and water. Subsequently, a corresponding amount of α-methacrylic acid (MAA) was added to the above solution based on a 1:4 molar ratio of the template molecule to the functional monomer, and ultrasonication was used to ensure that the two components were mixed thoroughly and homogeneously to form a pre-polymer, where the template molecule interacted with the functional monomer via hydrogen bonding and π–π stacking down. Next, 2.5 mmol of the cross-linking agent ethylene glycol dimethacrylate (EGDMA) and 1 mmol of the polymeric monomer acrylamide (AM) were added to this mixture. To enhance the electrical conductivity, an appropriate number of multi-walled carbon nanotubes were introduced into the system, and ultrasonic treatment was used to further ensure the homogeneous dispersion of the materials and to effectively remove possible air bubbles. Afterwards, the initiators potassium persulfate (KPS) and 2,2′-Azobis(2-methylpropionitrile) (AIBN), as well as the co-catalyst N,N,N′,N′-tetramethylethylenediamine (TMEDA), were sequentially added to the solution. Finally, the entire mixture was subjected to ultrasonic treatment for 10 min to eliminate residual air bubbles, and nitrogen purging was employed for 15 min to remove oxygen, thus preventing unwanted oxidation reactions. After completion of the above steps, the prepared mixture was placed in a thermostatic water bath at 60 °C for 24 h. The mixture is thermally activated at 60 °C, the initiator comes into play and the polymerization reaction takes place. This process was designed to achieve efficient synthesis of the target polymer by controlling the temperature and time conditions.

Upon completion of the polymerization process, the resultant product was initially rinsed with methanol to eliminate any unreacted monomers and other residual impurities. Subsequently, a mixed solvent with a volume ratio of methanol to acetic acid of 9:1 was used as the elution solvent to remove the template molecules. Then, the prepared molecularly imprinted hydrogel was dried using vacuum freeze-drying equipment to avoid structural collapse or unevenness that may result from direct drying.

#### 2.2.2. Preparation of the Molecularly Imprinted-Hydrogel Electrochemical Sensor

Before preparing the sensor, it was essential to preprocess the glassy carbon electrode (GCE). The preprocessing steps included gradually polishing the electrode with an α-alumina polishing powder of 1.0, 0.3, and 0.05 μm sizes. This was followed by ultrasonic cleaning in the sequence of ultrapure water, ethanol, and then ultrapure water again, and finally, nitrogen blowing to ensure that the electrode surface was clean and contaminant-free [23]. After completion of the preprocessing, CV was utilized with a scanning rate of 50 mV/s for 10 cycles to polymerize dopamine hydrochloride onto the surface of the glassy carbon electrode. Subsequently, the electrode surface was rinsed with ultrapure water to remove unpolymerized dopamine hydrochloride and other impurities. Next, carboxylated multi-walled carbon nanotubes, which had been pre-dissolved in a 50% ethanol solution at a concentration of 1 mg/mL, were modified onto the electrode surface by drop-coating with 5 µL. Once the electrode surface was dried, the prepared molecularly imprinted hydrogel was dissolved in methanol solution, and 5 µL was pipetted onto the glassy carbon electrode surface for modification. After the surface had dried, the prepared sensor was placed in a 0.1 mol/L [Fe (CN)_6_]^3−/4−^ solution for electrochemical testing.

#### 2.2.3. Electrochemical Testing Conditions

The electrochemical characterization was conducted in a solution containing 0.1 mol/L [Fe (CN)_6_]^3−/4−^, with specific methods including CV and Electrochemical Impedance Spectroscopy (EIS) measurements. The scan rate for CV was set at 100 mV/s, covering 8 segments, with a sampling interval of 1.0 mV for each segment. The EIS measurement was performed within a frequency range from 0.01 Hz to 100.0 Hz, with an applied AC signal amplitude of 5.0 mV. For performance analysis, DPV was used, with a potential range set from −0.2 V to 0.4 V, a pulse amplitude of 50.0 mV, a pulse width of 0.05 s, and a sampling width of 0.0167 s.

#### 2.2.4. Sample Processing

Weigh 2 g of the sample, add 1 mL of water, and shake thoroughly for 10 min. Subsequently, introduce 5 mL of acetonitrile and shake well for another 10 min. Then, add 4 g of anhydrous magnesium sulfate and 1 g of anhydrous sodium sulfate, and shake vigorously for 1 min. Centrifuge the mixture at 6000 rpm for 10 min. After centrifugation, transfer the supernatant to a clean container for storage, and document it as Part A. Add 4 mL of acetonitrile to the remaining solid residue and shake well to ensure thorough mixing. Then, add 4 g of anhydrous magnesium sulfate and 1 g of anhydrous sodium sulfate. Shake vigorously for 1 min, centrifuge at 6000 rpm for 10 min, and collect the supernatant after centrifugation. Collect the supernatant following centrifugation and document as Part B. Combine Part A and Part B, and transfer 6 mL of the resultant solution into a new centrifuge tube. Shake the mixture for 1 min and subsequently centrifuge at 6000 rpm for 5 min. Extract 1 mL of the supernatant and swiftly dry using a nitrogen stream. Subsequently re-dissolve the resulting dried residue in 1 mL of methanol. Employ a filter membrane to eliminate any particulate matter that might have been present.

## 3. Results and Discussion

### 3.1. Optimization of Experimental Conditions

**Figure 2:**
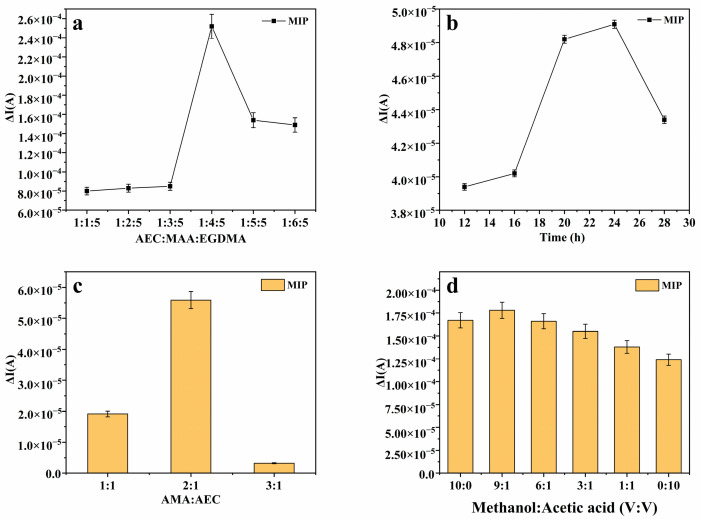
(**a**) Current response changes of molecularly imprinted hydrogels prepared with different molar ratios of template molecules, functional monomers, and cross-linking agents. (**b**) The influence of aggregation time on the change in sensor current response. (**c**) The influence of different molar ratios of polymer monomers and template molecules on current response. (**d**) Elution conditions: Changes in current response of the sensor after elution of the template with methanol and acetic acid in different volume ratios.

In order to achieve excellent sensing performance, the preparation conditions of molecularly imprinted hydrogels were optimized. This experiment primarily focused on optimizing the ratio of the template molecule to functional monomer and cross-linking agent, polymerization time, the amount of polymerization monomer added, and elution conditions. The optimization results are presented in Figure 2: the molar ratio of the template molecule to the functional monomer and cross-linking agent was refined to 1:4:5 (Figure 2a); the polymerization duration was optimized to 24 h (Figure 2b); the molar ratio of the polymerization monomer to the template molecule was adjusted to 1:2 (Figure 2c); and the chosen elution solution was a blend of methanol and acetic acid, with a volume ratio of 9:1 (Figure 2d).

**Figure 3:**
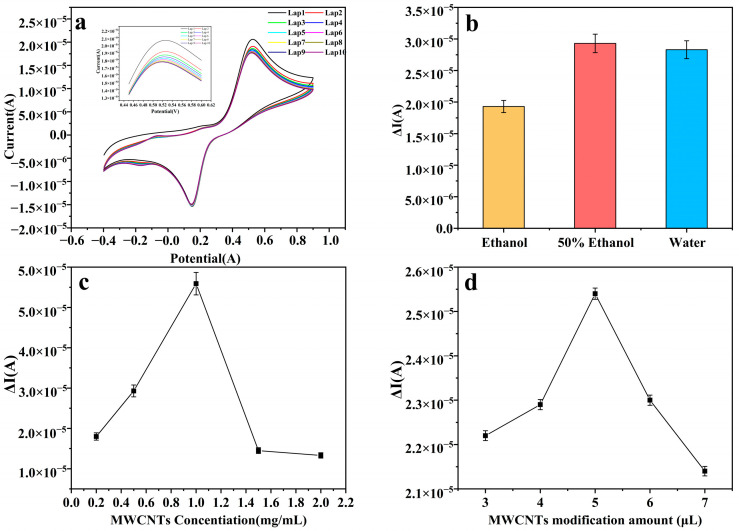
(**a**) CV diagram of dopamine hydrochloride electropolymerization. (**b**) The changes in CV current response values of MWCNT modified electrodes dissolved in different solvents. (**c**) The influence of MWCNT concentration on the change in sensor current response. (**d**) The effect of MWCNT modification on the change in sensor current response.

Furthermore, to enhance the sensitivity of the sensor, pretreatment of the glassy carbon electrode was carried out. Initially, a polydopamine layer was deposited on its surface via electropolymerization to bolster the electrode’s adhesion. The polymerization of dopamine hydrochloride on the glassy carbon electrode was successfully achieved via CV at a scan rate of 50 mV/s over 10 cycles, as illustrated in Figure 3a. To further enhance the electrode’s conductivity, we proceeded to process the conductive material, specifically multi-walled carbon nanotubes. These nanotubes were dissolved in a 50% ethanol solution, as shown in Figure 3b, to create a dispersion with a concentration of 1 mg/mL, depicted in Figure 3c. Subsequently, 5 μL of this dispersion was applied to the pretreated glassy carbon electrode, as evidenced in Figure 3d. All pertinent data are comprehensively presented in Figure 3.

**Figure 4:**
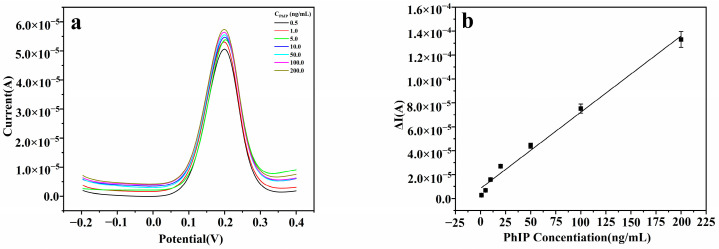
(**a**) DPV current response at different concentrations of PhIP. (**b**) Standard curve of DPV current difference and PhIP series concentration.

To assess the performance of the imprinted sensor, the current responses to varying concentrations of PhIP were measured under optimized experimental conditions. Figure 4a illustrates the trend of increasing current response with the elevation in PhIP concentration. Further analysis revealed that within the concentration range of 1 ng/mL to 200 ng/mL, a strong linear relationship exists between the current change and the PhIP concentration, as depicted in Figure 4b. This linear correlation can be quantified by the following linear regression equation: Δi = 8 × 10^−5^ × C_PhIP_ + 1 × 10^−5^ (R^2^ = 0.9931). Additionally, the sensor’s detection limit is 0.07 ng/mL (signal-to-noise ratio S/N = 3).

### 3.2. Characterization

**Figure 5:**
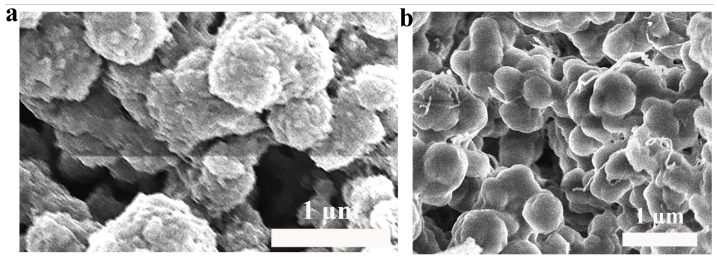
SEM images of molecularly imprinted hydrogels (**a**) before elution and (**b**) after elution.

The morphology of molecularly imprinted hydrogels before and after elution was examined via Scanning Electron Microscopy (SEM). Figure 5a, captured at a magnification of 11.45 KX, presents the SEM image of the molecularly imprinted hydrogel before elution. This image illustrates uneven pores, which arise from the imprinting sites created upon the combination of the template molecule AEC with the functional monomer MAA. In comparison, Figure 5b, shown at the same magnification of 11.45 KX, exhibits a more homogeneous surface with more consistently shaped pores. This change is due to the removal of template molecules during the elution process, resulting in pores that align with the shape and size of the template molecules. These imprinting active sites are conducive to the selective detection of PhIP. Since the polymerized MIH hydrogel maintains suitable rigidity under a specific degree of cross-linking, this rigidity supports the stability of the pores, thus guaranteeing the consistency and accuracy of the sensing reaction. As a result, the fabricated molecularly imprinted hydrogel enhances the sensing performance of PhIP, offering high selectivity, sensitivity, and stability.

**Figure 6:**
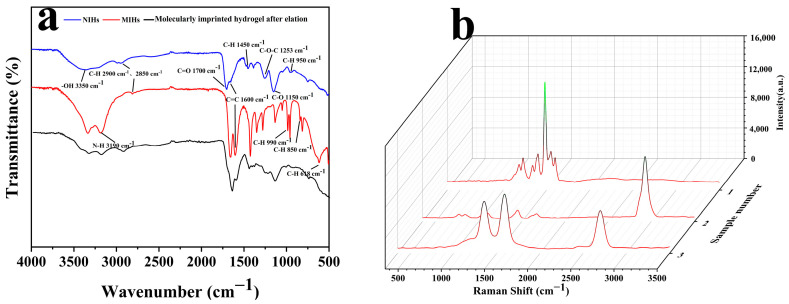
(**a**) Fourier transform infrared spectra of NIHs, MIHs, and eluted MIHs. (**b**) Raman spectra of NIHs, MIHs, and eluted MIHs.

Fourier transform infrared spectroscopy (FT-IR) verified the participation of functional monomers and cross-linking agents in the synthesis of molecularly imprinted hydrogels. In the FT-IR spectrum of Figure 6a, the peak at 1700 cm^−1^ corresponds to the carbonyl C=O absorption peak in MAA and EGDMA, while the peak at 1253 cm^−1^ corresponds to the C-O-C absorption peak in EGDMA. These observations indicate the successful synthesis of both molecularly imprinted and non-molecularly imprinted polymer layers. Additionally, the peak at 3190 cm^−1^ corresponds to the N-H stretching vibration of the AEC molecule, signifying that the AEC molecule has successfully combined with the functional monomer to form a specific recognition site. This further confirms the successful formation of the molecularly imprinted hydrogel. The presence of the peak at 1150 cm^−1^ also signifies that MAA has effectively engaged in the polymerization process, thereby reinforcing the confirmation of the molecularly imprinted hydrogel’s formation. From Figure 5, it is evident that following elution, the peak at 3190 cm^−1^ exhibits a notable reduction, signifying the successful removal of the template molecule AEC and confirming the effectiveness of the elution process. Furthermore, post-elution, the peaks at 990 cm^−1^, 950 cm^−1^, and 850 cm^−1^ also diminish, as they primarily arise from the C-H bending vibrations within the benzene ring of AEC molecules.

From the Raman spectra depicted in Figure 6b, it is evident that sample 1 corresponds to NIHs. This sample exhibits peaks at 1230, 1394, 1481, and 1538 cm^−1^, which are exclusively attributed to the polymer matrix (C-N, C-O-C, and C-H bending vibrations). Notably, these peaks lack the characteristic features of AECs, indicating that all observed peaks originate solely from the polymer. All peaks are from the polymer matrix, which is consistent with the characterization of non-imprinted materials. Sample 2 shows peaks at 836, 1099, 1445, 1568, and 2927 cm^−1^, of which 836 cm^−1^ may be related to the deformation vibration of the imidazole ring of AEC, 1568 cm^−1^ corresponds to the in-plane bending vibration of the aromatic ring of AEC, 2927 cm^−1^ corresponds to the C-H stretching vibration of the methyl group, and no peaks were found in the range of 3300–3500 cm^−1^. The absence of a peak in the 3300–3500 cm^−1^ range might be attributed to the C-H vibrational modes of the polymer matrix, which could interfere with the signal in this region, thereby obscuring the N-H peak. However, the presence of AEC is corroborated by the emergence of peaks at 1568 and 2927 cm^−1^. The peak observed at 1587 cm^−1^ for sample 3 corresponds to the target PhIP, indicating that the chemical environment within the cavity is conducive to recognizing PhIP. Additionally, the peak at 1353 cm^−1^ suggests that the heterocyclic complementary site may be appropriate for the imidazolium ring of PhIP. The absence of the N-H peak further confirms the efficient removal of the AEC. Although the eluted MIHs were not in direct contact with PhIP, the IR and Raman spectra together indicate that our prepared sensor has structural complementarity with the target.

### 3.3. Characterization of Adsorption Performance

**Figure 7:**
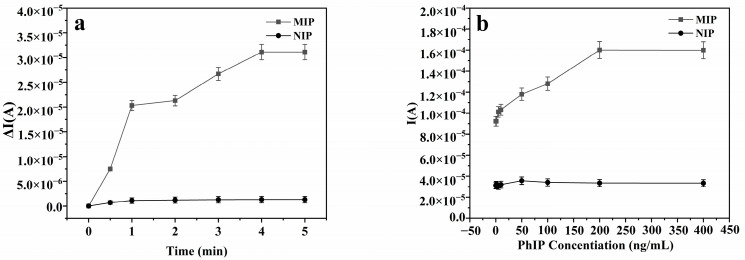
(**a**) Dynamic adsorption: changes in current response over time. (**b**) Static adsorption: fixed time, current response varying with target PhIP concentration.

To ascertain the duration needed for the sensor to achieve the adsorption equilibrium, we conducted a comprehensive investigation into its dynamic adsorption performance. In a solution with a concentration of 50 ng/mL of PhIP, we separately introduced MIHs–GCEs and NIHs–GCEs and monitored the alterations in the DPV response current. As depicted in Figure 7a, the most significant alteration in the current response was observed when the adsorption duration extended to 4 min. Subsequently, despite any additional extension of the adsorption time, the current response remained fundamentally unaltered, signifying that the sensor had attained the dynamic adsorption equilibrium at the 4 min mark.

Next, to determine the extent to which PhIP can be adsorbed to achieve equilibrium within the specified time frame, we maintained a constant adsorption duration and conducted experiments with varying concentrations of PhIP (1, 5, 10, 50, 100, 200, and 400 ng/mL). According to Figure 7b, when the concentration of PhIP reached 200 ng/mL, the current response change peaked. Despite any subsequent increases in PhIP concentration, the current response remained largely stable, signifying that the sensor had achieved the adsorption equilibrium at this concentration.

### 3.4. Selectivity, Stability, and Reproducibility

**Figure 8:**
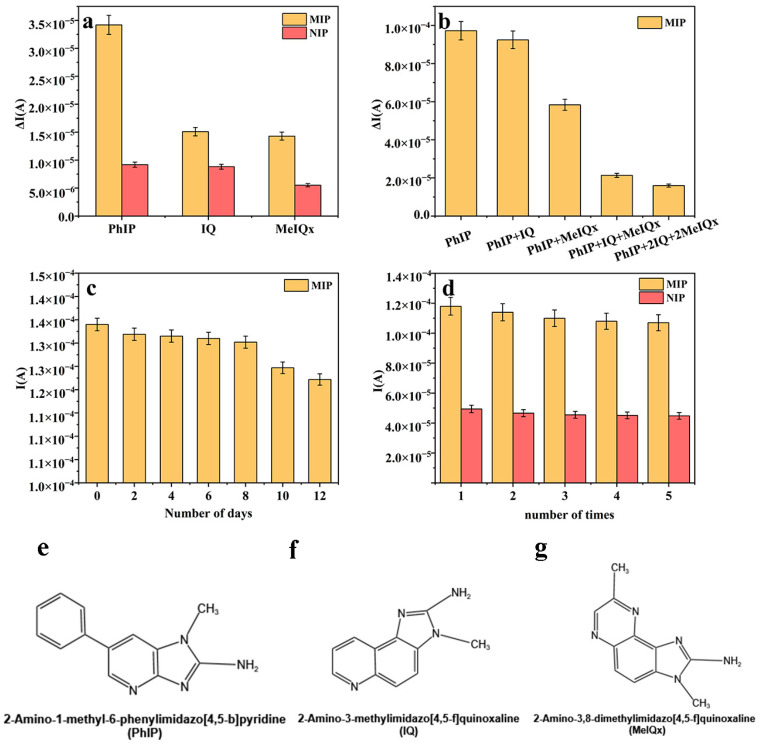
(**a**) The variation in current in a single-analyte standard experiment. (**b**) Mixed-analyte standard-experiment current variation value. (**c**) Daytime precision: the current variation in the same electrode over time. (**d**) Daily precision: measuring current changes 5 times on the same electrode. (**e**) Structural formula for PhIP. (**f**) Structural formula for IQ. (**g**) Structural formula for MeIQx.

To assess the recognition performance of molecularly imprinted hydrogel-modified glassy carbon electrodes towards the target molecule PhIP, we performed selectivity experiments by comparing the electrode’s response with that of structural analogs of PhIP, specifically 2-Amino-3-methylimidazo[4,5-f] quinoline (IQ) and 2-Amino-3,8-dimethylimidazo[4,5-f] quinoxaline (MeIQx). IQ is one of the heterocyclic amines that are produced when protein foods are heated, and it is considered to be carcinogenic [24]. (MeIQx) is one of the most abundant heterocyclic aromatic amines (HAAs) found in the human diet and is produced mainly during the high-temperature cooking of meat or fish [25]. Among them, the structural formulae of PhIP, IQ, and MeIQx are shown in Figure 8e–g. All substances were measured at a concentration of 30 ng/mL. As is depicted in Figure 8a,b, PhIP demonstrated the most significant current response change in both single-analyte and mixed-analyte experiments. This result underscores the prepared imprinted sensor’s excellent selectivity for PhIP recognition. The sensor’s selectivity is primarily due to the compatibility between the structure of PhIP and the imprinted site, along with the existence of non-covalent interactions.

To assess the stability of the sensor, it was kept in a refrigerator at 4 °C for a duration of 12 days. The results indicated that the current response, after a duration of 12 days, exhibited a mere decrease of 8.81% relative to the initial day, accompanied by a relative standard deviation of 3.05%. This result highlights the excellent stability of the prepared imprinted sensor (Figure 8c); however, to confirm its reproducibility, we also conducted five consecutive measurements of a 30 ng/mL PhIP solution using the same sensor, achieving a relative standard deviation of 4.09%. This demonstrates that the imprinted sensor is reusable and well-suited for the accurate determination of PhIP (Figure 8d).

### 3.5. Applicability of the Method

To evaluate the practicality of the imprinted sensor, we employed it to test samples of roasted chicken and roasted duck meat. As is illustrated in Table 1, we performed recovery experiments by analyzing PhIP-spiked samples at three distinct concentrations (1, 10, and 20 ng/mL) to evaluate the sensor’s performance. The results revealed that for the roasted chicken samples, the sensor’s recovery rate varied from 95.0% to 108.8%, with a relative standard deviation (RSD) of less than 9.68%. In the case of the roasted duck meat samples, the recovery rate ranged from 75.9% to 94.4%, with an RSD of less than 9.90%.

To further confirm the sensor’s practical applicability, we validated the prepared samples through High-Performance Liquid Chromatography (HPLC). The HPLC detection outcomes revealed that the recovery rate for the roasted chicken samples varied from 94.6% to 111.8%, with a relative standard deviation (RSD) of no more than 8.38%. In the case of the roasted duck meat samples, the recovery rate ranged between 77.00% and 96.17%, with an RSD of less than 9.49%. In addition, we compared the sensor with other analytical methods, as shown in Table 2, and although in some cases the recovery of the blotting sensor was slightly lower than that of HPLC and other methods, the preprocessing steps of the sensor were simpler and did not require complex equipment. Thus, the creation of this sensor provides a new method for the rapid electrochemical detection of heterocyclic amines, especially PhIP, in meat products in the field.

## 4. Conclusions

In this study, molecularly imprinted hydrogels were successfully synthesized via radical polymerization. These hydrogels were subsequently drop-coated onto the surfaces of glassy carbon electrodes to create PhIP molecularly imprinted electrochemical sensors. The experimental results revealed that the sensor exhibited excellent linearity within the concentration range of 1.0 ng/mL to 200 ng/mL, featuring a low detection limit of 0.07 ng/mL, and demonstrated outstanding selectivity for PhIP. When utilized for detecting roasted chicken samples, the sensor achieved spiked recovery rates ranging from 95.0% to 108.8%, with a relative standard deviation (RSD) of less than 9.68%. Although this work has achieved significant advancements in the rapid detection of PhIP, several challenges and opportunities necessitate further investigation. Firstly, in the realm of multi-analytical detection, there is a need to enhance the sensor’s capability to simultaneously detect other hazardous heterocyclic amines, such as IQ and MeIQx, through techniques like multi-template imprinting or array-based sensing platforms. Regarding long-term stability, it is crucial to assess the sensor’s performance under prolonged storage and repeated-use conditions to ensure its reliability in industrial settings. Additionally, efforts should be directed towards miniaturizing electrochemical workstations into portable devices for field testing, as well as developing simplified data-analysis interfaces for non-expert users. These initiatives aim to bridge the gap between laboratory-scale innovations and practical, real-world applications in food safety, thereby contributing to global efforts in carcinogen surveillance.

## Figures and Tables

**Table 1:** Testing of real samples (n = 3).

Detection Method	Electrochemical Workstation			High-Performance Liquid Chromatography		
	Scalar Addition (ng/mL)	Recovery Rate (%)	RSD(%)	Scalar Addition (ng/mL)	Recovery Rate (%)	RSD(%)
Grilled chicken	1	95.00	9.68	1	94.60	8.25
	10	103.70	5.77	10	110.20	8.38
	20	108.80	1.95	20	111.80	7.34
Roast duck meat	1	85.70	7.54	1	87.59	4.90
	10	75.90	9.90	10	77.00	9.49
	20	94.40	2.33	20	96.17	6.29

**Table 2:** Comparison of molecularly imprinted-hydrogel electrochemical sensors with other PhIP assays.

Analytical Methods	Test Sample	Linear Range (ng/mL)	Recovery Rate (%)	Reference
HPLC–Q-Orbitrap-HRMS	Pork, Beef	0.1–100.0	71.3–114.8%	[26]
HPLC–DAD–ESI-MS/MS	Pork, Beef, Chicken, Fish	20–6250	/	[27]
MSPE–HPLC–MS/MS	Bread, cake, French fries	0.5–100.0	98.2–108.2%	[28]
Molecularly Imprinted-Hydrogel Electrochemical Sensor	Grilled chicken, Roast duck meat	1.0–200.0	75.9–108.8%	This experiment

## Data Availability

The original contributions presented in this study are included in the article. Further inquiries can be directed to the corresponding author.
